# Cutaneous alpha‐synuclein deposition in postural tachycardia patients

**DOI:** 10.1002/acn3.51347

**Published:** 2021-03-25

**Authors:** Todd D. Levine, Bailey Bellaire, Christopher Gibbons, Roy Freeman

**Affiliations:** ^1^ Honor Health Neurology Scottsdale Arizona USA; ^2^ CND Life Sciences Phoenix Arizona USA; ^3^ Department of Neurology Beth Israel Deaconess Medical Center Harvard Medical School Boston Massachusetts USA

## Abstract

**Objective:**

To report a case series of patients with neuropathic POTS and cutaneous phosphorylated alpha‐synuclein (P‐SYN) deposition on skin biopsy and compare these to neuropathic POTS patients without P‐SYN deposition.

**Methods:**

The medical history, physical examination findings, autonomic function testing, and skin biopsy neuropathology of patients under the age of 50 with a postural tachycardia and a diagnosis of POTS were retrospectively reviewed. Included patients completed the composite autonomic severity score (COMPASS 31), the Wood Mental Fatigue Inventory, the Epworth Sleepiness scale, the REM Behavior Disorder Questionnaire, the Patient‐Reported Outcomes Measurement Information System (PROMIS‐10), and the Gastroparesis Cardinal Symptom Index.

**Results:**

Of 296 patients seen with POTS, 22 patients with suspected neuropathic POTS had skin biopsies performed during their evaluation. Seven of 22 patients had P‐SYN present on skin biopsy, while 15 individuals did not. Those with P‐SYN on biopsy: (1) were more likely to be male; (2) had features of REM sleep behavioral disorder; (3) reported less sleepiness and cognitive impairment; and (4) noted greater symptoms of gastroparesis. On autonomic testing, the group with P‐SYN deposition was more likely to have a hypertensive response to tilt‐table testing and abnormal QSART responses.

**Interpretation:**

Phosphorylated alpha‐synuclein deposition is present in some postural tachycardia patients with neuropathic features. Individuals with a postural tachycardia and cutaneous phosphorylated alpha‐synuclein deposition may be distinguished from other patients with neuropathic POTS.

## Introduction

A postural tachycardia is a non‐specific manifestation of orthostatic intolerance and may be seen in clinical settings that include volume depletion, dehydration, fever, anemia, and deconditioning. It is the cardinal feature of the postural orthostatic tachycardia syndrome (POTS), a heterogeneous disorder characterized by orthostatic intolerance and tachycardia while in the upright position, and accompanied by a constellation of symptoms and signs referable to multiple organ systems. POTS is considered the most common form of orthostatic intolerance with an estimated prevalence of 0.1–1% of the US population, although limited epidemiologic evidence exists.^1^, ^2^ POTS is defined as a heart rate increase of 30 beats/min or more within 10 min of head‐up tilt for those 20 years of age and older.^3^ POTS is more frequent in women than men (4.5:1)^1^, ^4^ and is most common between the ages of 15 and 50 years. A family history may be present in approximately 13% of patients.^4^, ^5^

Arguably, the subgroup of POTS in which the pathophysiology is best delineated is neuropathic POTS.^2^ In this subgroup, it is hypothesized that the postural tachycardia represents a restricted autonomic neuropathy, predominantly affecting the distal sympathetic innervation to the dependent vasculature, sparing the more proximal sympathetic cardiac innervation, thereby maintaining cardiac output and blood pressure by cardiac compensation. When this compensation fails, orthostatic hypotension ensues. From this perspective, neuropathic POTS can be positioned on the orthostatic intolerance continuum that ranges from postural tachycardia to delayed orthostatic hypotension to classical orthostatic hypotension.^3^

The notion that a neuropathic process may underlie the presentation of some patients with a postural tachycardia has prompted clinical investigations and yielded associations with disorders that are recognized causes of an autonomic and/or small fiber neuropathy.^6^ Furthermore, in some patients with neuropathic POTS, intraepidermal nerve fiber density is decreased, providing objective support for a small fiber neuropathy in these POTS patients.^7^

Misfolded alpha‐synuclein is the pathological hallmark of a group of disorders collectively known as the alpha‐synucleinopathies. Autonomic dysfunction is the cardinal feature of these disorders that include pure autonomic failure, Parkinson’s disease, dementia with Lewy bodies, and multiple system atrophy.^8^ A growing body of work from several laboratories has demonstrated the diagnostic utility of identifying cutaneous alpha‐synuclein in these disorders,^9^, ^10^, ^11^, ^12^, ^13^, ^14^, ^15^ however, to our knowledge, there are no reports of alpha‐synuclein deposition in patients with a postural tachycardia or POTS.

In this manuscript, we report a series of patients who underwent skin biopsy testing for evaluation of neuropathic POTS, and in whom phosphorylated alpha‐synuclein was identified within cutaneous nerve fibers.

## Patients and Methods

We retrospectively reviewed the medical history, physical examination findings, autonomic function testing, and skin biopsy neuropathology of patients under the age of 50 who presented to the Honor Health Neurological Service over 1 year with a postural tachycardia and a possible diagnosis of POTS. This study included patients with clinical symptoms consistent with POTS of at least 6 months duration. All patients had a complete medical history, including concomitant medication use, and a complete neurological and medical examination to exclude known causes of tachycardia. All patients with a known cause of their autonomic symptoms and tachycardia were excluded (e.g., diabetes, autoimmune disease, medications, thyroid disfunction, etc.). The study was approved by the Honor Health IRB.

All patients underwent autonomic function testing including head up tilt‐table testing to 60 degrees after a period of supine rest, heart rate variability in response to deep respiration, and a Valsalva maneuver. Patients also completed quantitative sudomotor axon reflex testing (QSART) at the foot, distal leg, distal thigh, and forearm (WR Medical, Maplewood, MN). Some patients with suspected neuropathic POTS had punch skin biopsies performed as part of their clinical evaluation. Three 3 mm skin punch samples were collected at the following sites: 10 cm above the lateral malleolus, 10 cm above lateral knee, and 3 cm lateral to the C7 vertebral prominence. Skin punch samples were processed according to previously published methods.^14^, ^15^ Briefly, tissues were fixed in Zamboni fixative, frozen and cut into 50 μm sections, and stained with immunofluorescent antibodies against protein gene product (PGP9.5) and phosphorylated alpha‐synuclein (P‐syn).^14^, ^15^ A total of six tissue sections for each of the three biopsies was analyzed (18 tissue sections reviewed per patient).

All patients completed the composite autonomic severity score (COMPASS 31),^16^ the Wood Mental Fatigue Inventory,^17^ the Epworth Sleepiness scale,^18^ the REM Behavior Disorder (RBD) Questionnaire,^19^ the Patient‐Reported Outcomes Measurement Information System (PROMIS‐10),^20^ and the Gastroparesis Cardinal Symptom Index.^21^

The results are reported by descriptive statistics, with mean ± standard deviation reported unless otherwise noted. Results were categorically analyzed by the presence, or absence, of phosphorylated alpha‐synuclein on skin biopsy, with results compared by Fishers exact test, unpaired t‐test, or Kruskal–Wallis testing if results were not normally distributed. Pearson correlations were used to describe relationships between tests. A *p* value of <0.05 was considered significant. Bonferroni corrections were made for multiple comparisons. Statistical analysis completed by SPSS 20 (SPSS, IBM Inc).

## Results

### Patient characteristics

**Figure 1:**
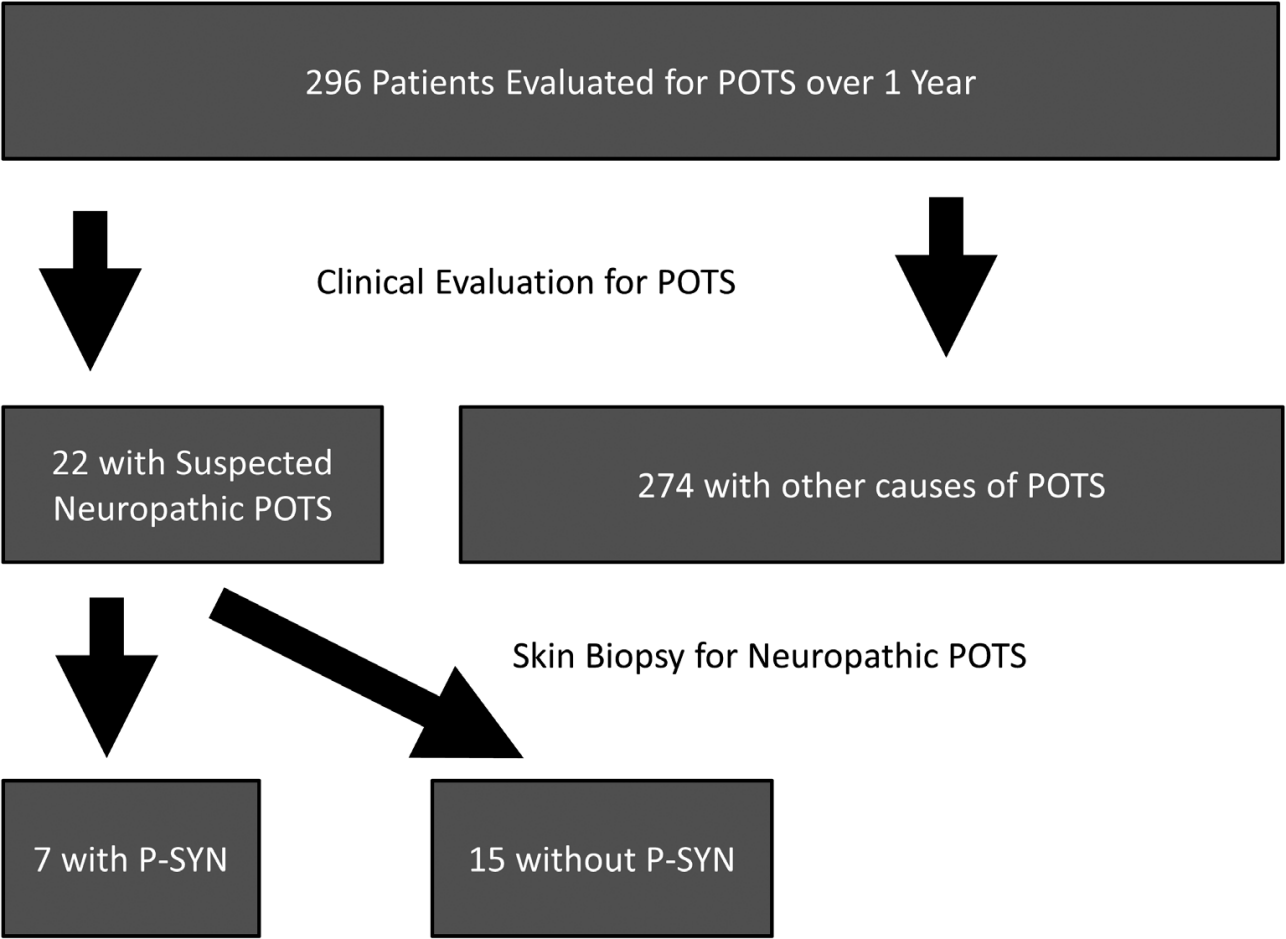
Consort diagram: The flow of the patients through the study is shown, from initial evaluation to post‐biopsy results. P‐SYN, phosphorylated alpha‐synuclein.

Of 296 patients referred to the neurology practice for the evaluation of POTS during a 1‐year period, we identified 22 patients who met consensus criteria for POTS and completed skin biopsies for evaluation of neuropathic POTS (Fig. 1: Consort Diagram). Patients were not included in the study (*N* = 274) if they had identifiable causes of tachycardia (such as medication effects or known underlying disease), incomplete medical records, did not have clinical features suggestive of neuropathic POTS, or did not complete a skin biopsy. Of the 22 POTS patients included in this study, the mean age was 32 years; and 17/22 (77%) were women. Three participants had a family history of a possibly synucleinopathy. The father of one individual with p‐SYN had a history of REM behavioral disorder. The father and paternal aunt of a second p‐SYN patient had multiple episodes of syncope that were thought to be due to a dysautonomia. The mother of one patient without P‐SYN had features of dysautonomia (gastroparesis and pacemaker placement for bradyarrhythmias) (See Table 1).

**Table 1:** Demographic information.

Demographic detail	P‐SYN positive	P‐SYN negative	*p* values
Number	7	15	
Age	31.1 (22–47)	32.7 (19–46)	NS
Gender (% female)	43%	93%	<0.05*
Duration of symptoms (years)	6.5 ± 5.1	6.4 ± 5.9	NS
Mean number of comorbid medical conditions	0.4	0.4	NS*
Family history of possible synucleinopathy	2/7	1/15	NS*
History of thermal dysregulation	1/7	4/15	NS*
History of resting tremors	0/7	3/15	NS*
History of anosmia	0/7	1/15	NS*
History of cognitive impairment or confusion	0/7	8/15	<0.05*
History of a sleep disorder	5/7	0/15	<0.001*
History of gastroparesis	5/7	0/15	<0.001*
History of constipation	6/7	4/15	NS*
History of bladder dysfunction	2/7	4/15	NS*

Significance measured by unpaired *T*‐test unless otherwise denoted (*Significance measured by Fisher’s exact test).

### Skin biopsy results

Seven of 22 patients had phosphorylated alpha‐synuclein present on skin biopsy, while 15 individuals did not. The intra‐epidermal nerve fiber density (IENFD) was similar in those with and without P‐syn, although there was a trend for those with P‐syn on biopsy to have skin biopsy results consistent with a small fiber neuropathy (4/7 cases had reduced IENFD) versus those with normal synuclein staining in whom only 2/15 had reduced IENFD (*p* = 0.08).

**Figure 2:**
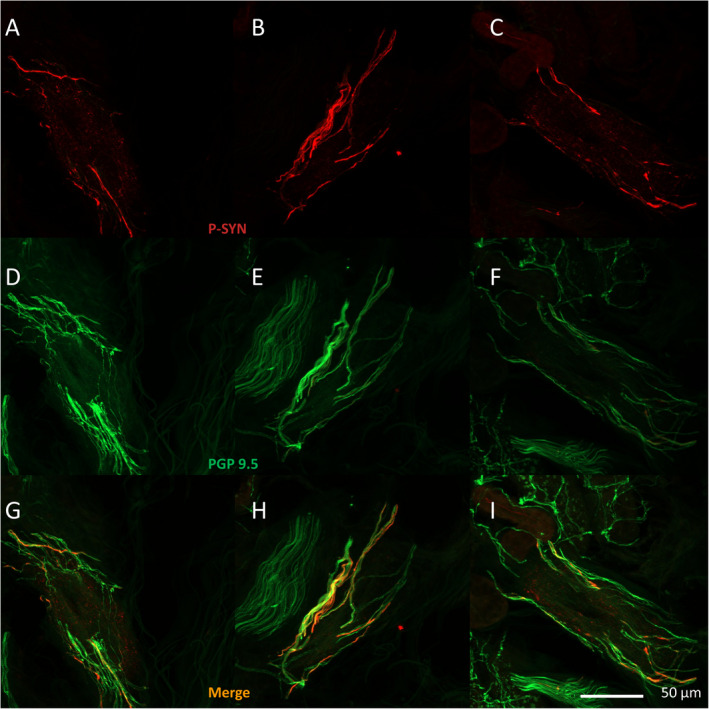
Sample images of cutaneous phosphorylated alpha‐synuclein. (A–I) Examples from three different patients with POTS of a blood vessel with surrounding vasomotor nerve fibers with phosphorylated alpha‐synuclein shown in red (A–C) and vasomotor nerve fibers shown in green stained with protein gene product 9.5 (D–F), overlapping merged images in (G–I).

P‐syn was detected in the posterior cervical biopsy in 5/7 cases, in the distal thigh biopsy in 4/7 cases, and the distal leg in 6/7 cases. In all 7 cases, P‐syn was detected within vasomotor nerve fibers (nerve fibers surrounding blood vessels). P‐syn was also detected within pilomotor nerve fibers, sudomotor nerve fibers, and nerve bundles (Fig. 2). In 5/7 cases, there were multiple nerve fibers that contained phosphorylated alpha‐synuclein in multiple skin biopsies. In 2/7 cases, only a single biopsy contained phosphorylated alpha‐synuclein.

### Demographic data and patient history

The complete demographic information for the two groups based on presence or absence of P‐syn is listed in Table 1. There were statistically more men in the P‐syn‐positive group compared to the P‐syn‐negative group (*p* < 0.05, Fishers exact test).

P‐SYN‐positive patients reported sleep‐related symptoms and symptoms suggestive of gastroparesis more frequently then P‐syn‐negative patients. Patients without P‐syn were more likely to report cognitive impairment or confusion. There were no significant differences in disease duration or number of comorbid medical conditions (See Table 1).

### Autonomic function testing

A summary of the autonomic testing results is shown in Table 2. Parasympathetic function (heart rate variability to deep breathing and the Valsalva ratio) was similar in the two groups. There were significant differences in sympathetic cholinergic function, as measured by QSART. In the group without P‐syn, 2/15 patients had one or more reduced QSART responses. In contrast, the group with abnormal P‐syn had 6/7 patients with at least one abnormal QSART response (*p* < 0.05, Fishers exact test). The hemodynamic responses during tilt‐table testing revealed a similar heart rate increase in the two groups. There was a significant difference in blood pressure response to tilt. The group without evidence of phosphorylated alpha‐synuclein had a modest increase in blood pressure during tilt‐table testing (9 ± 8 mmHg) compared to group with P‐syn where there was a greater increase in blood pressure during tilt (32 ± 17 mmHg, *p* < 0.01). The baseline heart rate change in heart rate, highest heart rates, and other changes in blood pressure were similar between the two groups.

**Table 2:** Autonomic function.

Test	P‐SYN positive	P‐SYN negative	*p* values
Heart rate deep breathing (ratio)	1.29 ± 0.13	1.3 ± 0.12	NS
Valsalva ratio	2.37 ± 0.44	2.01 ± 0.49	NS
Baseline heart rate (beats per minute)	72 ± 13	78 ± 8	NS
Highest heart rate during 10‐min tilt	106 ± 14	111 ± 9	NS
Change in heart rate during tilt	33.7 ± 4.3	31.7 ± 3.9	NS
Baseline SBP (mmHg)	121 ± 13	117 ± 12	NS
Highest SBP during tilt (mmHg)	153 ± 23	125 ± 14	<0.01
Delta SBP change during tilt (mmHg)	32 ± 17	9 ± 8	<0.01
Baseline DBP(mmHg)	74 ± 7	71 ± 7	NS
Delta DBP change during tilt (mmHg)	23 ± 13	15 ± 8	NS
Highest DBP during tilt (mmHg)	97 ± 15	86 ± 11	NS
QSART abnormal (percent of patients)	86%	13%	<0.05*
IENFD distal leg	11.4 ± 3.6	11.4 ± 4.6	NS
IENFD distal thigh	15.8 ± 4.4	17.5 ± 3.7	NS
IENFD posterior cervical	30.0 ± 3.7	29.9 ± 6.1	NS

Significance measured by unpaired *T*‐test unless otherwise denoted (*Significance measured by Fisher’s exact test).

### Patient‐reported questionnaires

**Figure 3:**
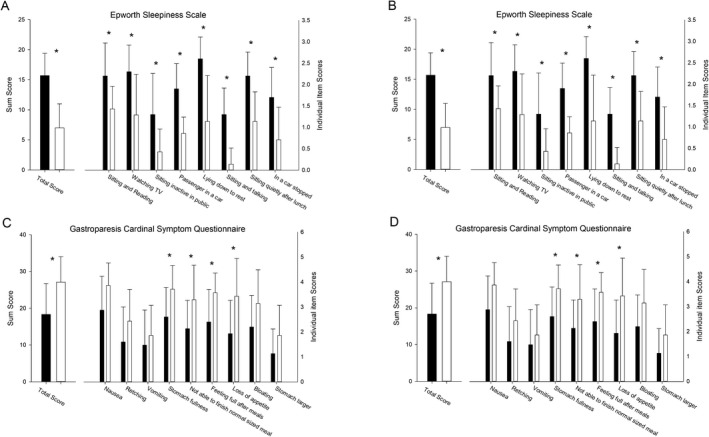
(A–D) Results of the patient reported outcome questionnaires. (A) REM sleep behavioral disorder questionnaire. The individual item responses are shown on the left *Y* axis, with the total score shown on the right *Y* axis. *p < 0.01. (B) Gastroparesis Cardinal Symptom Index. The individual item responses are shown on the left Y axis, with the total score shown on the right Y axis. *p < 0.01. (C) Epworth Sleepiness Scale. The individual item responses are shown on the left Y axis, with the total score shown on the right *Y* axis. *p < 0.01. (D) Results from the Wood Mental Fatigue Inventory. The individual item responses are shown on the left *Y* axis, with the total score shown on the right *Y* axis. *p < 0.01.

The results of the individual questionnaire data are reported in Table 3 and Figure 3A–D. The individuals with P‐syn on skin biopsy reported more symptoms of REM sleep behavioral disorder and gastroparesis. In contrast, individuals without P‐syn reported more symptoms of sleepiness and mental fatigue.

**Table 3:** Symptom questionnaires.

Questionnaire	P‐SYN positive	P‐SYN negative	*p* values
Epworth sleepiness scale (Scale 0–24)	7.0 ± 4.0	15.7 ± 3.7	<0.001
Gastroparesis cardinal symptom questionnaire (Scale 0–45)	27.1 ± 8.3	18.3 ± 10.4	<0.05
COMPASS 31 (Scale 0–76)	26.1 ± 9.4	32.5 ± 13.3	NS
RBD questionnaire (Scale 0–10)	3.7 ± 1.9	0.1 ± 0.3	<0.005
Mental fatigue Scale (Scale 0–45)	14.0 ± 10.3	32.2 ± 6.2	<0.005
Promis 10 (Scale 0–50)	25.3 ± 10.1	21.9 ± 6.2	NS

Significance measured by unpaired *T*‐test.

#### REM sleep behavioral disorder questionnaire

The RBD questionnaire total score was higher in the P‐syn‐positive patients. Significantly different questionnaire items included vivid dreams, arms and legs moving during sleep, hurt myself or bed partner, and movements awaken me (Fig. 3A).

#### Gastroparesis cardinal symptom index

The Gastroparesis questionnaire total score was higher in the P‐syn‐positive patients. Significantly different questionnaire items included stomach fullness, feeling full after meals, and loss of appetite (Fig. 3B).

#### Epworth sleepiness scale

The sleepiness scale total score was higher in the P‐syn‐negative patients. Significantly different questionnaire items included sleepiness while sitting and reading, while watching TV and while a passenger in a car (Fig. 3C).

#### Woods mental fatigue inventory

The mental fatigue scale total score was higher in the P‐syn‐negative patients. Significantly different questionnaire items included poor concentration, difficulty with memory, difficulty making decisions, feeling “muzzy” headed, and slow thoughts (Fig. 3D).

#### Autonomic questionnaire and quality of life

There were no significant differences in responses between groups using the COMPASS‐31 autonomic questionnaire or the Promis‐10 quality‐of‐life questionnaire (See Table 1).

## Discussion

We report a case series of individuals with a diagnosis of POTS who have evidence of phosphorylated alpha‐synuclein deposition on punch skin biopsies. Of 296 patients referred for the evaluation of POTS over a 12‐month period, 22 patients had features consistent with neuropathic POTS and had skin biopsies as part of their work up. Seven of 22 patients had cutaneous alpha‐synuclein deposition detected on skin biopsy. This group of patients showed important demographic and phenotypic differences compared to the POTS group without P‐SYN and the general POTS population. Specifically, of the group with P‐SYN‐positive biopsies, men were more common than in the POTS group without P‐SYN. This is in contrast to most studies of POTS that have a 5:1 or greater female:male ratio.^5^, ^22^ In addition, based on results from validated patient‐reported symptom questionnaires, important phenotypic differences were present in the P‐SYN group compared with the POTS group without P‐SYN. These included: (1) features of RBD were significantly more common; (2) sleepiness and cognitive impairment were significantly less common; and (3) symptoms of gastroparesis were significantly more common. Finally, on autonomic testing, the group with P‐SYN deposition was more likely to have a hypertensive response on the tilt‐table^23^ and abnormal QSART responses.^7^ Taken together, this study suggests that P‐SYN deposition may be present in some postural tachycardia patients with neuropathic features, and that individuals with a postural tachycardia and cutaneous phosphorylated alpha‐synuclein deposition may be distinguished from other patients with POTS.

Several lines of evidence support the concept that neuropathic POTS in some individuals is due to a restricted peripheral neuropathy; specifically, sympathetic denervation predominantly in the lower hemibody with preservation or relative preservation of cardiac sympathetic innervation. The evidence includes reports of venous denervation,^24^ impaired distal sudomotor function,^25^, ^26^ lower norepinephrine spillover in the legs than the arms,^27^ quantitative sensory test results,^28^ and the association with denervation on skin biopsy.^7^, ^29^ Estimates suggest that in tertiary referral center studies, a neuropathic etiology may underlie POTS in 33 to 50% of individuals.^2^, ^4^, ^27^ These data have prompted a search for a specific neurological cause in POTS patients and possible etiologies that have been uncovered include diabetes,^30^ Sjogren’s syndrome,^31^ and autoimmune autonomic ganglionopathy.^32^ The results of the present study should be viewed in this context, specifically, that alpha‐synuclein deposition may underlie the postural tachycardia and accompanying symptoms in a subset of patients.

Skin biopsy with assessment of intra‐epidermal nerve fiber density has become the gold‐standard diagnostic test for the assessment of small fiber neuropathy. More recently, the technique has expanded to encompass the assessment of cutaneous deposition of misfolded proteins including alpha‐synuclein. Data from multiple centers using similar techniques support the diagnostic utility of this assessment in the alpha‐synucleinopathies, pure autonomic failure,^11^ idiopathic Parkinson’s disease, dementia with Lewy bodies and multiple system atrophy,^13^ and REM behavioral disorder.^33^, ^34^ The test characteristics include high reproducibility with a sensitivity of 80–95% and a specificity of >95% even in patients with early‐stage Parkinson’s disease (Hoehn and Yahr stages I and II) and prodromal conditions such as idiopathic REM sleep disorder.^14^, ^33^, ^35^

The spectrum of neurogenic orthostatic intolerance extends from classical orthostatic hypotension, to delayed orthostatic hypotension to neuropathic POTS. Viewed from this perspective, in individuals with neuropathic POTS, in the face of attenuated systemic vascular resistance due to distal sympathetic denervation, blood pressure is maintained by a compensatory tachycardia that increases cardiac output. Whereas in patients with classical neurogenic orthostatic hypotension, the homeostatic reflexes that maintain blood pressure fail due to central and/or peripheral neurodegeneration. We initially proposed that delayed orthostatic hypotension, which was associated with mild abnormalities of sympathetic adrenergic function, may be a mild or early manifestation of sympathetic autonomic failure.^36^ In a follow‐up study of that cohort, 54% of individuals with delayed OH progressed to classical OH and 31% of individuals with delayed OH developed an a‐synucleinopathy.^37^ Thus, it is not surprising that some patients with a milder form of orthostatic intolerance, that is, neuropathic POTS, have an alpha‐synucleinopathy. In contrast to the longitudinal study of delayed OH, longitudinal studies of patients with POTS support a good outcome in the most but not all patients.^4^ The presence of cutaneous alpha‐synuclein may be a biomarker for some of those patients who have a less favorable outcome but this requires further study.

The clinical phenotype of the postural tachycardia patients with alpha‐synuclein deposition was significantly different to that of the POTS group without P‐SYN.^38^ Of the sleep‐related symptoms in POTS patients, fatigue, sleepiness, and unrefreshing sleep are among the most prevalent. In the present study, consistent with prior studies, symptoms related to sleep were common in both groups. However, sleepiness, measured using the Epworth Sleepiness Scale, was significantly more severe in the POTS group without P‐SYN. Not only was the overall score significantly worse but all individual items of the scale were significantly worse in those without P‐SYN on biopsy. In contrast, RBD symptoms, although not highly prevalent in studies of POTS patients, were reported commonly in the alpha‐synuclein‐positive group. The defining polysomnographic feature of RBD is increased electromyographic tone with or without abnormal behaviors during REM sleep. None of our patients have had polysomnogram studies, however, the P‐SYN‐positive group had a significantly higher overall score on the RBD screening questionnaire compared to the POTS group without P‐SYN, and most individual items of the questionnaire were significantly worse in the P‐SYN‐positive group.

Several longitudinal studies have documented that patients with idiopathic RBD are at high risk of neurodegenerative disease, most commonly a synucleinopathy.^39^, ^40^, ^41^ The largest and longest duration of these studies suggests a phenotype conversion rate of 6.25% per year, with 73.5% of the cohort developing a neurodegenerative disease, most commonly an alpha‐synucleinopathy, over 12 years of follow up.^41^ The implications of finding the RBD symptom constellation in a postural tachycardia cohort are not known, and this group, in particular, requires longitudinal follow‐up.

Upper gastrointestinal symptoms, suggestive of gastroparesis, were prominent in the P‐SYN‐positive group who had a significantly higher total score on the validated Gastroparesis Cardinal Symptom Index compared to the POTS group without P‐SYN. Gastrointestinal symptoms are common in the synucleinopathies and many studies have documented synuclein deposition throughout the gastrointestinal tract.^42^, ^43^ However, symptoms in the earliest disease stage—and most studies refer to Parkinson’s disease—are usually referable to the lower gastrointestinal tract.^42^, ^43^ Indeed, constipation is a prominent premotor symptom in epidemiologic studies of Parkinson’s disease and may antedate motor symptoms by >20 years.^43^, ^44^ In contrast, gastroparesis is characteristically a late manifestation in Parkinson’s disease, although, paradoxically, in pathological studies, the distribution of alpha‐synuclein deposition is highest in the proximal gastrointestinal tract and lowest in the rectum and colon.^45^ The relationship between this observation and the symptoms in the P‐SYN patients will require further study. In contrast, in studies of POTS patients, upper gastrointestinal symptoms consistent with gastroparesis are highly prevalent, particularly in patients with neuropathic POTS.^46^

POTS patients report a broad range of cognitive difficulties.^47^, ^48^ This constellation of complaints, colloquially termed “brain fog”, is reported to occur in the seated and supine position and may affect selective cognitive domains on standardized neuropsychological testing.^48^ While both groups endorsed some individual items on the Wood Mental Fatigue Inventory, a patient self‐report inventory, the POTS control group had an inventory total score similar to that reported in a previous POTS study.^49^ In contrast, the P‐SYN group had a significantly lower total score and significantly lower scores on all individual items of the inventory reinforcing the notion that this is a phenotypically different group, and in particular, a group with fewer cognitive complaints.

Orthostatic hypertension, a rise in blood pressure during tilt or stand, was seen in the group of individuals with P‐SYN on skin biopsy. Orthostatic hypertension has previously been reported as an early, or attenuated form of autonomic dysfunction in patients with POTS, norepinephrine transporter deficiency, baroreflex dysfunction, and central autonomic dysregulation.^50^ The association between orthostatic hypertension and P‐SYN deposition is not known.

The diagnostic approach and conclusions that underlie the present manuscript are consonant with classical illness nosological principles, that is, syndromes such as POTS, Sjogren’s syndrome, Tourette’s syndrome, and essential tremor, for example, are defined as a recurring group of clinical symptoms, signs, and test results without a known etiology.^51^, ^52^, ^53^ Inherent to these principles is that a defined syndrome is a waystation^51^—a “diagnostic placeholder”—on the scientific journey toward the discovery of specific etiologies and treatment. Thus, the diagnostic approach and identification of cutaneous alpha‐synuclein in these patients could be considered a step toward the delineation of an etiology in a small subgroup of the heterogeneous postural tachycardia syndrome population.

These findings should be viewed with an abundance of caution. Although the data support an association between a postural tachycardia with neuropathic features and cutaneous alpha‐synuclein deposition in a very small group of patients, this is a case–control study and longitudinal data are not available Thus, it is not known whether these findings are an early stage in the progression toward one of the clinically evident alpha‐synucleinopathies—pure autonomic failure, Parkinson’s disease, dementia with Lewy bodies, and multiple system atrophy—or merely an incidental finding. In addition, referral bias is likely; the data emanate from a single practice with a special interest in peripheral neuropathy, thus, it seems likely that these findings are of relevance only to a small number of individuals with a postural tachycardia in the general population, even those with features suggesting a neuropathic cause of the postural tachycardia. Viewed in the context of all POTS patients referred for evaluation, the prevalence of cutaneous alpha‐synuclein deposition was only 2.3%. Nevertheless, although these results and their implications should be studied in a larger population with longitudinal follow‐up, the present findings in a select group of patients, provide a potential explanation for one of the earliest manifestations of autonomic failure in patients with a syndrome that remains poorly understood.

## Conflict of Interest

The authors have stock options in CND Life Sciences (T.L., C.G., and R.F.) or are employees of CND Life Sciences (B.B.). CND Life Sciences performs synuclein staining on skin biopsies.

## Funding Information

No funding information provided.
